# Beyond Recanalization: A Mechanistic and Procedural Framework for Adjunct Pharmacologic Therapy During Mechanical Thrombectomy

**DOI:** 10.3390/jcm15176681

**Published:** 2026-08-28

**Authors:** Saniyah Shaikh, Touleen T. Raslan, Affaf Tanweer, Zainab Nasir, Hibba Siraj, Thaabit Raziq, Umaima Shoukat, Volodymyr Mavrych, Olena Bolgova, Ahmed Yaqinuddin

**Affiliations:** 1 College of Medicine, Alfaisal University, Riyadh 11533, Saudi Arabia; sanshaikh@alfaisal.edu (S.S.); traslan@alfaisal.edu (T.T.R.); atanweer@alfaisal.edu (A.T.); znasir@alfaisal.edu (Z.N.); hsiraj@alfaisal.edu (H.S.); mraziq@alfaisal.edu (T.R.); vmavrych@alfaisal.edu (V.M.); obolgova@alfaisal.edu (O.B.); 2 SVS Medical College, Mahbubnagar 509001, India; umaimasara2006@gmail.com

**Keywords:** acute ischemic stroke, endovascular thrombectomy, adjunct pharmacotherapy, reperfusion injury, microvascular dysfunction

## Abstract

Endovascular thrombectomy [EVT] has revolutionized acute ischemic stroke care by restoring macrovascular reperfusion in large vessel occlusion; however, a persistent “reperfusion–outcome gap” remains, leaving nearly half of successfully recanalized patients without functional independence. This review aims to bridge this translational gap by establishing a mechanistic and procedural framework for adjunctive pharmacotherapy designed to target the microvascular, thromboinflammatory, and reperfusion-related injuries left unaddressed by purely mechanical models. Synthesis of recent literature reveals that futile recanalization is driven by three distinct anatomical and biological bottlenecks: distal microembolization, microcirculatory no-reflow, and ischemia–reperfusion injury. Furthermore, the inconsistency of clinical trial results reflects a critical precision deficit, including dilution of treatment effects across heterogeneous reperfusion grades, inadequate stroke etiology stratification, delayed pharmacologic intervention relative to evolving microvascular injury, and reliance on functional endpoints that inadequately capture tissue-level reperfusion. To address these limitations, this review outlines a tri-axial clinical decision framework integrating reperfusion status, stroke etiology and hemorrhagic risk, which maps specific patient phenotypes, such as incomplete macrovascular reperfusion or underlying intracranial atherosclerotic disease, to targeted pharmacologic strategies including microcatheter-directed intra-arterial thrombolytics and glycoprotein IIb/IIIa inhibitors. The review further argues that future progress will require imaging-guided identification of residual hypoperfusion, mechanistically enriched patient enrolment, and incorporation of biological and tissue-level reperfusion endpoints alongside conventional functional outcomes. Ultimately, closing the reperfusion–outcome gap will require moving beyond empirical, universal drug administration toward a precision, pharmacologically augmented EVT paradigm that aligns adjunctive therapy with mechanism-specific procedural phenotypes and prioritizes true tissue-level recovery over angiographic success alone.

## 1. Introduction

Endovascular thrombectomy [EVT] is a minimally invasive treatment for acute ischemic stroke, particularly in patients with large vessel occlusion. Its introduction has changed the management of these strokes and is now considered a major advance in cerebrovascular care. Several landmark randomized trials established EVT as the standard of care for eligible patients by showing better reperfusion rates and improved functional outcomes compared with medical therapy alone [1]. However, even though mechanical thrombectomy achieves successful recanalization in more than 80–90% of patients, this does not always result in meaningful clinical recovery [2]. Only about half of these patients regain functional independence at 90 days, demonstrating that the clinical discrepancy between angiographic success and neurological recovery still remains [1,3,4].

The “reperfusion-outcome gap”, also known as futile recanalization, has gained growing recognition due to this discrepancy [5]. This concept depicts the failure to achieve significant neurological recovery despite technically successful recanalization of large vessels [3,5]. While EVT effectively restores macrovascular blood flow, a significant number of patients continue to experience suboptimal clinical outcomes such as infarct progression, neurological deterioration, or severe long-term disability even after achieving favorable modified Thrombolysis in Cerebral Infarction [mTICI] scores—subsequently refined as the expanded TICI [eTICI] scale used throughout this review [3,6]. Recent evidence shows that optimal clinical recovery and tissue-level perfusion cannot be accomplished through angiographic recanalization alone, rendering the reperfusion–outcome gap a core challenge in modern stroke intervention [4,6].

Distal embolization is one of the primary factors contributing to this phenomenon. Mechanical manipulation of thrombi may fragment clot material, creating distal microemboli that obstruct downstream blood vessels beyond the resolution of standard angiographic visualization tools. Consequently, clot fragmentation stands as a major determinant of incomplete reperfusion and ischemic injury despite successful vessel recanalization [3]. Another important contributor is the persistent microcirculatory failure despite restoration of proximal arterial patency, also known as the microvascular no-reflow phenomenon [4]. The no-reflow phenomenon is characterized by a complex sequela of interconnected ischemic microvascular dysfunction, including endothelial swelling, pericyte constriction, leukocyte adhesion, platelet aggregation, microthrombosis, and capillary compression. These microvascular changes prevent adequate downstream tissue perfusion even when angiographic reperfusion appears successful. Recent studies suggest that no-reflow may affect approximately 25–30% of EVT-treated patients, with estimates varying by definition [angiographic eTICI vs. perfusion imaging, detailed in Section 3.2] and is independently associated with progressive tissue injury and poor neurological outcomes [7,8,9].

Residual thrombus is considered an underrecognized limitation of EVT. Even after a technically successful endovascular thrombectomy retrieval of large proximal clots, residual thrombotic material may continue to obstruct distal arterial branches or the microcirculation. This issue contributes to incomplete tissue perfusion and continued ischemic injury and may promote early vessel re-occlusion [3]. Recent observational and clinical studies suggest adjunct intra-arterial thrombolytic therapy as a potential approach to target thrombotic material beyond the reach of mechanical devices alone, as it may improve microvascular reperfusion without significantly increasing hemorrhagic risk [10].

Simultaneously, reperfusion injury has become widely recognized as a major biological consequence of successful vessel reopening. The sudden restoration of blood flow into previously ischemic vessels triggers the neurovascular inflammatory cascade, thus potentially exacerbating neuronal injury. Therefore, this proves that ischemia–reperfusion injury plays a major role in futile recanalization, highlighting that reperfusion itself may be biologically harmful when inadequately modulated [2,4].

Importantly, futile reperfusion should be distinguished from poor outcome resulting primarily from irreversible ischemic injury established before reperfusion. Mechanism-directed adjunctive therapies are therefore most relevant when viable or potentially salvageable tissue remains but tissue-level perfusion remains inadequate despite successful macrovessel recanalization. Time to reperfusion, baseline infarct burden, collateral status, and post-recanalization tissue perfusion should consequently be considered when attributing poor outcome to modifiable mechanisms of futile reperfusion [3,4,6,11,12]. These mechanisms should not be interpreted as a fixed hierarchical sequence. Rather, they represent interconnected and potentially overlapping contributors to tissue-level reperfusion failure. Distal embolization can sustain downstream vascular obstruction, while microvascular no-reflow may arise from interacting processes including microthrombosis, endothelial dysfunction, pericyte contraction, and edema. Ischemia–reperfusion injury can further amplify these processes through inflammatory, oxidative, and BBB-disruptive pathways. Their relative contribution is therefore likely to vary according to thrombus characteristics, time to reperfusion, collateral status, baseline tissue injury, and the post-recanalization microvascular state.

Ultimately, these factors highlight the limitations of a purely mechanical model for stroke treatment, such as EVT. EVT is highly effective at reopening large occluded vessels, but successful recanalization does not necessarily mean that tissue-level perfusion has been fully restored [6]. Injury at the microvascular level can continue after the vessel has been reopened, partly because of thromboinflammation, endothelial dysfunction, and reperfusion-related damage [2]. For this reason, angiographic success alone may not explain why some patients recover well while others do not [4,5].

The next stage in acute ischemic stroke treatment may involve combining mechanical reperfusion with pharmacological therapies that act beyond the proximal clot [2,13]. While EVT focuses on reopening the occluded large vessel, additional treatments may help support the distal microcirculation and limit secondary injury after reperfusion [10,13]. This approach would not replace thrombectomy but rather complement it by addressing the microvascular and inflammatory processes that may continue after successful recanalization [3,13].

## 2. Materials and Methods

This study was conducted as a narrative review aimed at synthesizing mechanistic, procedural, and clinical evidence relevant to adjunct pharmacologic therapy during mechanical thrombectomy for acute ischemic stroke, with the objective of integrating these domains to develop a conceptual decision framework. A structured search strategy was used to ensure comprehensive coverage of the mechanistic and clinical literature; however, it was not conducted as a formal systematic review—no PRISMA flow diagram, dual-reviewer screening, or formal risk-of-bias assessment was performed, consistent with the conceptual/synthetic aims of a narrative review. The review was guided by the premise that adjunct pharmacologic therapies should be deployed according to mechanism-specific procedural phenotypes defined by reperfusion status, stroke etiology, and hemorrhagic risk.

A structured literature search was performed from inception through 7 May 2026, using PubMed/MEDLINE, PubMed Central [PMC], Scopus, the Cochrane Library, and Google Scholar. Searches employed combinations of Medical Subject Headings [MeSH] and free-text terms related to acute ischemic stroke, mechanical thrombectomy, adjunct pharmacotherapy, intra-arterial thrombolysis, glycoprotein IIb/IIIa inhibitors, distal embolization, microvascular no-reflow, reperfusion injury, intracranial atherosclerotic disease, hemorrhagic transformation, clot composition, and tissue reperfusion. Additional searches incorporated major clinical trials, including CHOICE, RESCUE BT, OPTIMISTIC, BRIDGE-TNK, and EXTEND-IA TNK.

Articles were included if they informed one or more of three domains central to this review: ref. [1] focused on the pathophysiologic mechanisms underlying the reperfusion outcome gap, including clot-level limitations, microvascular dysfunction, and reperfusion injury; ref. [2] provided evidence regarding adjunct pharmacologic strategies, including intra-arterial thrombolytics, antiplatelet agents, and emerging therapies; and ref. [3] showed the limitations of current clinical evidence, including patient selection, treatment timing, and outcome assessment. Evidence was prioritized according to study design and direct relevance to adjunct pharmacologic therapy during EVT, with randomized controlled trials and prospective comparative studies given greatest weight, followed by systematic reviews/meta-analyses and observational studies. Mechanistic, experimental, and consensus literature was used primarily to contextualize biological rationale and identify emerging therapeutic strategies. Eligible study designs included randomized controlled trials, observational studies, registries, systematic reviews, meta-analyses, experimental studies, expert consensus statements, and clinical guidelines. Only English-language publications were included. Studies restricted to mechanical thrombectomy without a pharmacologic adjunct or those focused solely on neuroprotection without relevance to the reperfusion–outcome gap were excluded.

The retrieved literature was synthesized thematically according to the manuscript’s conceptual structure. Evidence was integrated to develop a three-axis mechanistic–procedural framework based on reperfusion status, stroke etiology, and hemorrhagic risk, and applied across four clinical scenarios: incomplete reperfusion, distal embolization, intracranial atherosclerotic disease with re-occlusion risk, and elevated hemorrhagic risk. Two figures were developed iteratively by the author team to visually summarize the mechanistic targets of adjunct pharmacologic therapy and the proposed decision framework. The review emphasizes areas of convergent evidence, unresolved controversies, and future directions for precision endovascular therapy.

## 3. Pathophysiologic Basis for Adjunct Pharmacology

Failure of tissue recovery after successful thrombectomy reflects injury across three progressively smaller anatomical scales: residual thrombus burden, microcirculatory no-reflow, and reperfusion injury [3]. Each domain implies a distinct pharmacologic target, therapeutic window, and hemorrhagic risk calculus. Distinguishing these mechanisms is essential for rational adjunctive therapy design; empirical addition of agents to undifferentiated reperfusion populations has been the field’s principal translational failure mode. These mechanisms should not be interpreted as uniformly sequential or mutually exclusive. Their relative contribution likely varies according to stroke etiology, ischemic duration, collateral status, and timing and completeness of reperfusion, and substantial biological overlap may exist between these processes.

### 3.1. Clot-Level Limitations

Residual mural thrombus and emboli dislodged into distal branches during device retrieval likely contribute to sustained tissue-level hypoperfusion in territories that appear angiographically reperfused [14]. Intra-arterial [IA] fibrinolytics delivered at high local concentration through the indwelling microcatheter target this niche. The CHOICE trial demonstrated that IA alteplase after eTICI 2b50-3 reperfusion increased modified Rankin Scale [mRS] 0–1 from 40.4% to 59.0% without excess symptomatic intracranial hemorrhage [sICH] [15]. POST-TNK showed a directionally consistent but non-significant trend for IA tenecteplase [49.1% vs. 44.1%] with neutral safety [16]. Pooled randomized data converge on a risk ratio of approximately 1.23 [95% CI 1.11–1.36] for excellent outcome with no detectable sICH rise [17]. The benefit clustering at mRS 0–1 rather than broader disability endpoints may reflect preservation of functionally critical microterritories, though the trials were not designed to test this spatial hypothesis directly. The RITE registry supports a downstream embolic mechanism: rescue IA alteplase in patients with TICI ≥ 2A improved angiographic scores without functional gains, supporting the interpretation that adjunct fibrinolysis complements rather than substitutes for successful proximal recanalization [18].

Clot composition modulates therapeutic response along a gradient from fibrin-/platelet-rich thrombi, exhibiting viscoelastic stiffness and lysis resistance [19,20], to RBC-rich thrombi that fragment readily, require fewer retrieval passes, and show greater fibrinolytic sensitivity [14]. However, this binary framework incompletely captures thrombectomy behavior. The STRIP registry found no overall association between composition and first-pass effect [21], while higher fibrin content was associated with lower first-pass recanalization rates and the need for more retrieval passes, consistent with greater cohesion but reduced lytic and mechanical susceptibility [22]. Observational data suggest hemorrhagic risk may also vary by composition: any-grade intracranial hemorrhage [ICH] was 15.8% in RBC-dominant versus 1.7% in fibrin-dominant cases in one single-center cohort [23], though confounding by reperfusion completeness and retrieval-pass burden limits causal inference. Collectively, these findings indicate that thrombus composition influences thrombectomy behavior through competing effects on lytic susceptibility, mechanical cohesion, and embolic fragmentation. No trial has stratified IA thrombolysis by clot histology or its imaging surrogates, leaving the mechanism-to-therapy bridge inferential [14,19].

### 3.2. Microcirculatory Dysfunction

Macrovascular reperfusion does not ensure capillary reperfusion. Microvascular no-reflow—persistent capillary hypoperfusion despite upstream patency—likely mediates a substantial proportion of futile recanalization. Among the contributing mechanisms, platelet-rich microthrombi and neutrophil extracellular trap formation currently have the strongest translational evidence and therefore represent the most actionable therapeutic targets. Pericyte contraction, endothelial swelling, and astrocytic compression are biologically plausible contributors but remain less clinically validated [7,24]. Thus, the proposed hierarchy is not one of proven causal dominance but of current mechanistic and translational evidence, with thromboinflammatory microvascular obstruction representing the most clinically actionable target, followed by endothelial and cellular contractile mechanisms requiring further validation. Cerebral venous outflow may also influence tissue-level reperfusion after arterial recanalization, although its contribution to futile reperfusion and its therapeutic modifiability remain insufficiently established and therefore fall outside the primary arterial–microvascular framework considered here. Prevalence estimates range from less than 10% under strict eTICI 3 definitions to approximately 30% by perfusion imaging [8]—the two ends of the range reflect different case-ascertainment methods, not conflicting evidence, with persistent tissue-level hypoperfusion independently associated with loss of thrombectomy benefit [11]. This definitional heterogeneity complicates biomarker-guided trial enrichment.

A critical distinction separates territorial hypoperfusion—downstream emboli potentially responsive to fibrinolysis—from true capillary no-reflow, which requires microvascular-targeted therapy [7,24]. CHOICE imaging data support the interpretation that IA alteplase improves perfusion predominantly in eTICI 2b patients, consistent with embolic clearance rather than capillary rescue [15]. Glycoprotein IIb/IIIa inhibitors address platelet-driven mechanisms but have yielded heterogeneous results. Postprocedural dual antiplatelet strategies appear to outperform periprocedural bolus approaches in stented patients, though effect sizes remain modest [25]. Selective thromboinflammatory targets such as GPVI inhibition and TLR4 antagonism remain clinically unvalidated [7]. The persistent trial-signal gap likely reflects three factors: non-enriched enrollment, limited safe antithrombotic dosing, and delayed treatment after capillary obstruction is established [7,24].

### 3.3. Reperfusion Injury

Reperfusion triggers coordinated oxidative, inflammatory, and excitotoxic cascades that can convert the ischemic penumbra into infarction or hemorrhage within minutes to hours of recanalization [26]. Reactive oxygen species-mediated blood–brain barrier [BBB] degradation and matrix metalloproteinase activation produce hemorrhagic transformation; this is the principal safety constraint on adjunct pharmacology [23]. Antioxidant strategies including edaravone derivatives improve early surrogate endpoints but rarely translate to durable 90-day functional benefit [27,28]. This recurrent mismatch suggests that oxidative stress, while mechanistically central, is insufficient as a monotherapy target when parallel cascades remain active. Unlike clot-level or microvascular adjuncts, cytoprotective therapies operate under a substantially narrower temporal therapeutic window because injury cascades begin immediately after reperfusion. BBB-stabilizing agents, including imatinib, 3K3A-activated protein C, and SUR1 inhibitors, converge mechanistically on barrier preservation despite acting through distinct molecular pathways, but all remain at early clinical stages [29]. Reviews of recent cytoprotectant trials consistently identify conditional efficacy confined to mechanistically defined subpopulations, a pattern that underscores the futility of testing single agents in heterogeneous cohorts [28]. Stroke Therapy Academic Industry Roundtable [STAIR XIII] reframed prior neuroprotection failures as problems of biological mistiming and population heterogeneity rather than absence of mechanistic efficacy [30,31]. Pooled IA thrombolysis data provide partial reassurance, with no sICH excess across randomized trials [17]. This safety profile, however, does not justify indiscriminate pharmacologic addition.

### 3.4. Synthesis: From Mechanism to Procedural Phenotype

**Figure 1:**
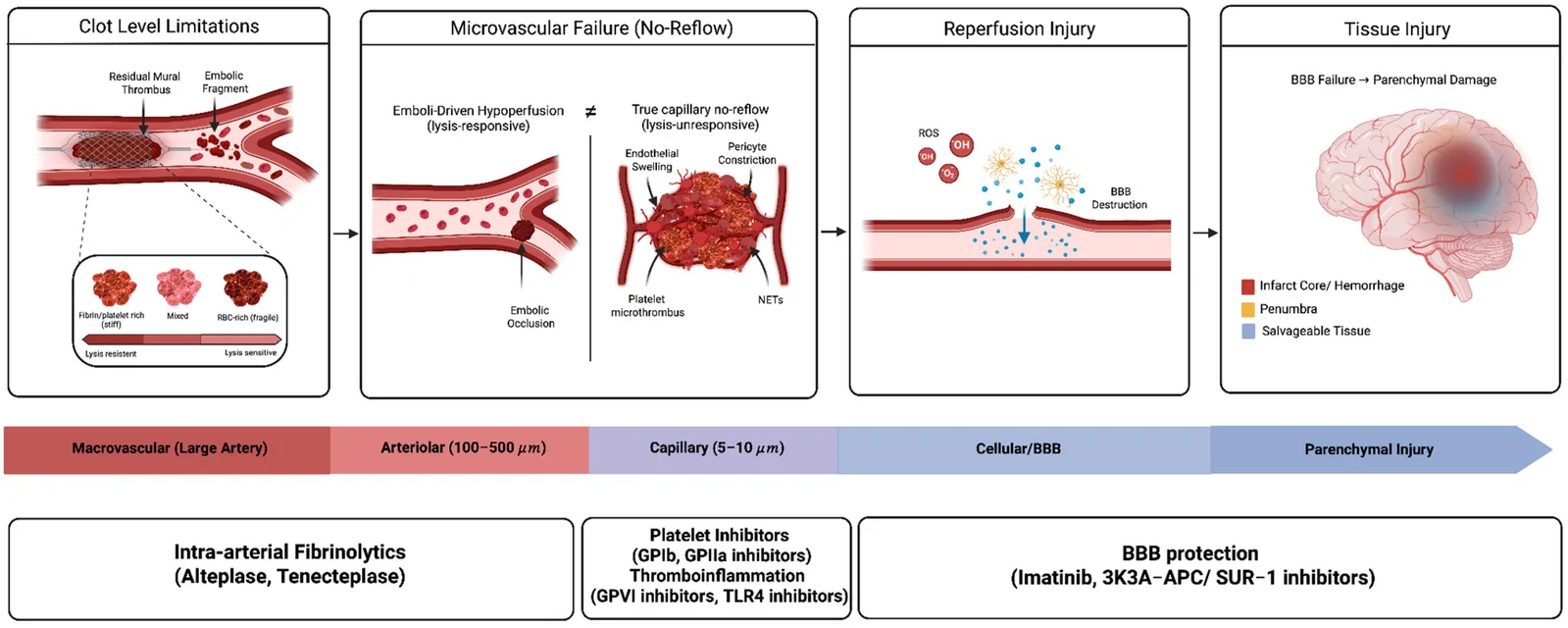
Mechanistic targets of adjunct pharmacologic therapy in EVT. Pathophysiologic cascade following large-vessel occlusion and mechanical thrombectomy. Clot fragmentation may cause distal embolization, leading to microvascular obstruction and no-reflow despite successful recanalization. Subsequent reperfusion can trigger inflammation, oxidative stress, edema, and hemorrhagic transformation. Adjunct pharmacologic therapies target these stages to improve tissue-level reperfusion and clinical outcomes.

These three domains form interconnected contributors along a single reperfusion cascade: clot clearance shapes macrovascular patency, microvascular function shapes tissue-level perfusion, and reperfusion-injury tolerance shapes parenchymal survival. Future progress will likely require mechanism-matched pharmacology deployed in imaging-defined subpopulations rather than empirical application across undifferentiated cohorts. The remainder of this review translates this framework into a procedural decision model stratified by reperfusion status, stroke etiology, and hemorrhagic risk [see Figure 1 for mechanistic targets].

## 4. Adjunct Pharmacologic Strategies: Evidence and Interpretation

Acute ischemic stroke management has been revolutionized by the introduction of endovascular mechanical thrombectomy [EVT]. Despite its enormous success in restoring large vessel patency, downstream microvascular mechanisms remain unaddressed. Therefore, adjunct pharmacological strategies have been encouraged to add to EVT’s success by addressing various complex biological processes such as distal embolization, residual thrombus, microcirculatory dysfunction, and thrombo-inflammatory injury, as these issues remain unresolved post-clot retrieval. In parallel, the potential value of these therapies appears highly dependent on procedural context and the underlying nature of the stroke rather than universal administration [3].

Intra-arterial thrombolysis [IA] has emerged as one of the most direct adjunctive pharmacological strategies to EVT. In patients with eTICI 2b reperfusion, where significant downstream hypoperfusion may persist despite successful proximal recanalization, IA fibrinolysis is justified, as thrombectomy often fails to clear distal microemboli or fully restore tissue-level perfusion [32,33]. The CHOICE randomized clinical trial included patients who received intra-arterial alteplase immediately after a technically successful mechanical thrombectomy. These patients had better functional outcomes than those in the placebo group, suggesting that adjunct thrombolysis after endovascular thrombectomy may improve microvascular reperfusion in a way that supports neurological recovery. Importantly, rates of symptomatic intracranial hemorrhage were not increased, which suggests that these benefits were achieved without an obvious safety trade-off [32]. Still, the findings should be interpreted cautiously, since the study included a relatively small and selected patient population [32,33]. Larger multicenter trials are needed to confirm whether the results apply more broadly [32]. The therapeutic benefit of adjunct intra-arterial thrombolysis is mostly demonstrated in TICI 2B patients, or those with physical clot substrate remaining that provides a target for the drug [33]. However, in patients who have achieved complete macrovascular recanalization e[TICI 3], and perfusion deficits remain, the likely underlying reasoning is due to inflammation or a secondary cause rather than a thrombus [3,33]. In similar cases, providing these patients with a clot-busting drug when no underlying clot is present is considered to be unnecessary and harmful to the patients’ safety and well-being by increasing the risk of bleeding [33]. This concept demonstrates that intra-arterial thrombolysis is not a one-size-fits-all intervention but a targeted strategy tailored to specific angiographic phenotypes [3,32]. However, various clinical limitations persist despite these promising outcomes. Trial heterogeneity, particularly regarding optimal drug dosages, infusion durations, and timing relative to thrombectomy, remains substantial [33]. Furthermore, existing studies’ sample sizes remain too small to determine the definitive hemorrhagic risk [32,33]. This demands larger, well-powered clinical trials to standardize these variables and determine safety boundaries.

Tirofiban is a glycoprotein IIb/IIIa inhibitor used as adjunctive antiplatelet therapy post-mechanical thrombectomy to prevent platelet adhesion and vessel reocclusion. This target strategy is especially successful in patients with underlying intracranial atherosclerotic disease (ICAD), as the clot removal process in similar cases often triggers focal endothelial injury and promotes new clot formation, leading to vessel reocclusion. Recent observational studies suggest that this intervention, when administered selectively, is associated with reduced re-occlusion rates and progressive early neurological stability. Hemorrhagic transformation, especially in patients with compromised blood–brain barrier integrity or an extensive history of large strokes, remains a core limitation of glycoprotein IIb/IIIa inhibition. Given the variability across studies in antiplatelet dosing, timing, and prior use of IV thrombolysis, larger randomized controlled trials are still needed before these findings can be applied more broadly. For now, adjunctive antiplatelet therapy should be reserved for carefully selected patients based on procedural findings and imaging features. This may include patients with residual clot fragments on angiography, suspected intracranial atherosclerotic disease, or cases requiring multiple thrombectomy passes. In this context, antiplatelet therapy is best viewed as a targeted option for specific stroke mechanisms rather than a routine add-on to improve reperfusion in all patients [3]. Emerging therapies are increasingly focused on the injury that occurs after mechanical recanalization, particularly ischemia–reperfusion injury and persistent microvascular dysfunction. This shift reflects the limited success of traditional neuroprotective agents in large randomized trials, which may be partly explained by poor treatment timing, inadequate patient selection, and limited integration with modern reperfusion therapies [34,35].

Current research has therefore moved toward anti-inflammatory and endothelial-targeted approaches [3,34]. After reperfusion, oxidative stress, leukocyte adhesion, complement activation, and endothelial injury can all contribute to ongoing tissue damage, secondary infarct expansion, blood–brain barrier disruption, and hemorrhagic transformation [34]. Because of this, there is growing interest in therapies that stabilize the neurovascular unit, protect the blood–brain barrier, and preserve microvascular tone [34,35].

These strategies may help address the gap between successful vessel recanalization and incomplete clinical recovery by targeting the microvascular injury that can persist after thrombectomy [3]. However, most of these agents remain investigational, and stronger clinical trial evidence is needed before they can be incorporated into routine practice [3,34].

In conclusion, therapeutic efficacy is largely dependent on its mechanistic context. Intra-arterial thrombolytics demonstrate their highest efficacy in patients who have a residual thrombus or a distal embolus [33]; antiplatelets such as glycoprotein IIb/IIIa inhibitors find their justification in ICAD-driven thrombosis [3], and emerging experimental treatment strategies are aimed at targeting tissue inflammation, such as the ischemia–reperfusion injury cascade [34]. Adjunctive pharmacological therapy is to be considered a tool in the new age of personalized medicine instead of empirical administration to all patients regardless of their procedural and biological phenotype [3]. Currently, however, these strategies lack the firm clinical evidence to mandate their inclusion in routine endovascular protocols [3,33]. Ultimately, the new frontier in stroke management should include a standard where pharmacological augmentation is selectively tailored to the underlying microvascular issue rather than being a universal blanket measure [3].

### 4.1. Why Current Evidence Remains Inconclusive Adjunct Pharmacologic Strategies

The persistent evidentiary gap for adjunct pharmacology in EVT reflects not an absence of biologic efficacy, but a systematic failure of investigative precision. Approximately 51% of patients undergoing EVT experience futile recanalization—functional dependence despite successful reperfusion—a proportion that has persisted despite successive improvements in device technology [36]. Four interrelated methodological shortcomings account for this gap: TICI-score dilution producing false-negative trials, insufficient stratification by stroke etiology, a timing paradox whereby pharmacologic delivery lags behind progressive microvascular injury, and a misalignment between the mRS endpoint and the tissue-level processes that adjunct therapy targets.

#### 4.1.1. The “False-Negative Trial” Problem

The most consequential structural flaw in adjunct pharmacology trials has been the co-enrollment of patients achieving complete reperfusion [eTICI 2c–3] alongside those with incomplete reperfusion [eTICI 2b50], conflating phenotypes with opposing pharmacologic requirements [16,37]. The POST-TNK trial, restricted to eTICI 2c–3 patients [*n* = 540], found no functional benefit from adjunctive intra-arterial tenecteplase [16]. The same neutral result was seen in POST-UK [37]. In contrast, the CHOICE trial, enrolling patients across eTICI 2b50–3, demonstrated significant improvement in excellent neurological outcome with intra-arterial alteplase [15]. At ISC 2025, both ANGEL-TNK and PEARL [enrolling eTICI ≥2b50] reported significant benefit with intra-arterial tenecteplase and alteplase, respectively [38,39]. A pooled meta-analysis of four RCTs found no significant benefit for the broader good functional outcome endpoint [mRS 0–2: OR 1.07; 95% CI 0.86–1.32], though the same analysis did demonstrate a significant benefit for excellent outcome [mRS 0–1: OR 1.31; 95% CI 1.06–1.63] [33]. The attenuated signal for good outcome may partly reflect dilution of a potentially pharmacologically responsive eTICI 2b50 subgroup by eTICI 2c–3 patients in whom adjunct therapy may confer limited or no incremental benefit.

#### 4.1.2. Heterogeneity in Patient Selection

Combining cardioembolic and ICAD-related etiologies under the same eligibility threshold produces attenuated and uninterpretable effect estimates. ICAD-related LVO mediated by in situ platelet-rich thrombosis on disrupted or stenotic atherosclerotic plaque mechanically responds to antiplatelet augmentation, whereas cardioembolic stroke is fibrin-dominated and carries higher hemorrhagic risk [40,41]. In a large multicenter cohort of LAA patients, tirofiban was associated with better functional outcomes in anterior circulation strokes compared to posterior circulation strokes [adjusted OR 2.163; 95% CI 1.130–4.140] [42]. A meta-analysis of over 11,700 EVT patients established cardioembolic etiology as an independent predictor of futile recanalization [OR 1.34; 95% CI 1.10–1.63] [36]. The methodological implication is clear: etiology-stratified enrollment is a prerequisite for testing any pharmacologic hypothesis in EVT.

#### 4.1.3. The Timing Paradox

Microvascular dysfunction begins within minutes of ischemic onset and progresses toward structural irreversibility with sustained ischemic duration. Pericyte contraction, endothelial swelling, neutrophil and platelet plugging, and fibrin microthrombosis—the cellular substrate of no-reflow—evolve along this continuum [43,44]. No-reflow has been documented in up to 30% of patients consistent with the perfusion-imaging-defined range in Section 3.2 after successful large-vessel EVT and is independently associated with poor outcomes. Current trial designs neither capture microvascular status at pharmacologic administration nor include it as a predictive biomarker, meaning agents are delivered to a microvascular environment that may already be beyond rescue [7,9]. Post-EVT CT perfusion data show residual hypoperfusion volume < 3.5 cc predicts favorable 90-day outcome [OR 3.5; 95% CI 1.6–7.8] [12], suggesting a defined imaging threshold may delineate the window within which adjunct pharmacology can alter tissue fate; a threshold overlooked by existing protocols.

#### 4.1.4. Endpoint Misalignment

The 90-day mRS condenses a heterogeneous range of neurological states into seven crude ordinal categories incapable of measuring microvascular and parenchymal responses. An intervention that reduces distal embolic burden and partially restores microvascular patency may convert a patient from mRS 1 to mRS 0; a clinically meaningful shift. However, most EVT trials define favorable outcome as mRS 0–2, making this shift entirely undetectable by the primary endpoint [45,46]. Imaging surrogates offer mechanistically superior alternatives: hypoperfusion intensity ratio [HIR], post-EVT Tmax > 6 s territory, and 24 h DWI infarct volume can directly measure microvascular reperfusion independent of macrovascular eTICI score [6,12,47]. Inflammatory biomarkers, including interleukin-6 and neutrophil count, both independently associated with futile reperfusion, provide additional pharmacodynamic endpoints that mRS cannot resolve [48,49].

#### 4.1.5. Core Argument: A Precision Problem That May Contribute to Apparent Efficacy Inconsistency

The divergence between neutral POST-TNK/POST-UK results [restricted to eTICI 2c–3] and positive CHOICE/ANGEL-TNK/PEARL results [enrolling eTICI ≥2b50] is precisely what a precision-based model predicts [15,16,37,38,39]. The inconsistency of existing evidence may reflect, in part, limitations in the precision of trial design rather than a uniform absence of efficacy. Mechanism-specific enrolment, etiology stratification, imaging-defined timing windows, and pharmacodynamically sensitive endpoints are not incremental refinements. They are the necessary scientific prerequisites to assess whether adjunct pharmacology can close the reperfusion–outcome gap. Continued testing of pharmacologic therapies in heterogeneous populations using coarse functional outcomes may therefore continue to obscure treatment effects that are restricted to biologically defined subgroups. Importantly, improved trial precision should not be assumed to guarantee a positive treatment effect. Some pharmacologic agents may have limited biological efficacy even within appropriately selected populations, and prospective trials are required to distinguish true treatment inefficacy from dilution of treatment effects by heterogeneous enrollment.

Operationally, each biological phenotype implies a distinct therapeutic target. Distal embolization and residual thrombus may warrant further clot-directed intervention, including selective intra-arterial thrombolysis when appropriate; microvascular no-reflow may require strategies aimed at restoring tissue-level perfusion and mitigating microvascular obstruction; and reperfusion injury may require approaches targeting blood–brain barrier dysfunction, inflammation, edema, and oxidative stress. These mechanisms should be assessed using complementary angiographic, perfusion, and structural imaging, supplemented where validated by circulating biomarkers, rather than by a single diagnostic measure [7,10,12,15,16,17,18,26,27,28,29,47]. Candidate biomarkers, including inflammatory mediators, endothelial injury markers, platelet-activation indices, and circulating markers associated with thrombotic activity, such as D-dimer, may provide additional biological information; however, their ability to distinguish the dominant mechanism of futile reperfusion and guide treatment selection remains insufficiently validated [24,48,49].

Although the present framework focuses primarily on arterial and microvascular determinants of tissue-level reperfusion, venous outflow may represent an additional contributor to cerebral perfusion and microcirculatory recovery after arterial recanalization. However, its specific contribution to futile reperfusion and its potential therapeutic modulation remain insufficiently defined. Venous mechanisms are therefore considered a potential complementary factor rather than a primary therapeutic axis of the present framework.

## 5. A Mechanistic–Procedural Decision Framework

### 5.1. Framework Principles

The continuous inconsistency observed between the angiographic recanalization and the real impact on functionality exhibits a structural defect of agnostic mechanistic rationale to adjuncts in the endovascular thrombectomy [EVT]. Instead of administering medication empirically or uniformly, it would be more appropriate to employ a structured decision-analysis framework that combines the procedural result and pathophysiological logic and specifics of the patient’s risk. We introduce a tri-axial model that enacts the central idea at the point of care.

Axis 1: Reperfusion Status [eTICI Grade]. eTICI 2b reperfusion [50–89% filling] indicates incomplete macrovascular restoration and a worse prognosis than those with eTICI 2c/3 [15]. Post-procedural perfusion imaging shows that a substantial proportion of patients with eTICI 2b reperfusion retain residual hypoperfusion, identifying the subgroup where adjunctive therapy has biological plausibility [12,15]. In this subgroup, the clearest benefit signal came from CHOICE [15,50].

Axis 2: Stroke Mechanism. Three mechanistic subtypes warrant distinct consideration. First, distal embolization results in clot beyond device reach; intra-arterial thrombolytics are mechanistically aligned [50]. Second, intracranial atherosclerotic disease [ICAD] reflects fixed stenosis, platelet-driven thrombosis, and high re-occlusion propensity; antiplatelet agents address this pathophysiology [51,52]. Third, microvascular primary failure lacks a proven pharmacologic effect.

Axis 3: Hemorrhagic Risk. Pharmacologic augmentation must be calibrated against the risk of hemorrhagic transformation. ASPECTS < 6, established infarct core, prior anticoagulation, age > 80, and prolonged time to recanalization are the primary predictors [51,52]. Patients at high risk should receive lower doses, intra-arterial delivery rather than intravenous, or no adjunct therapy.

**Figure 2:**
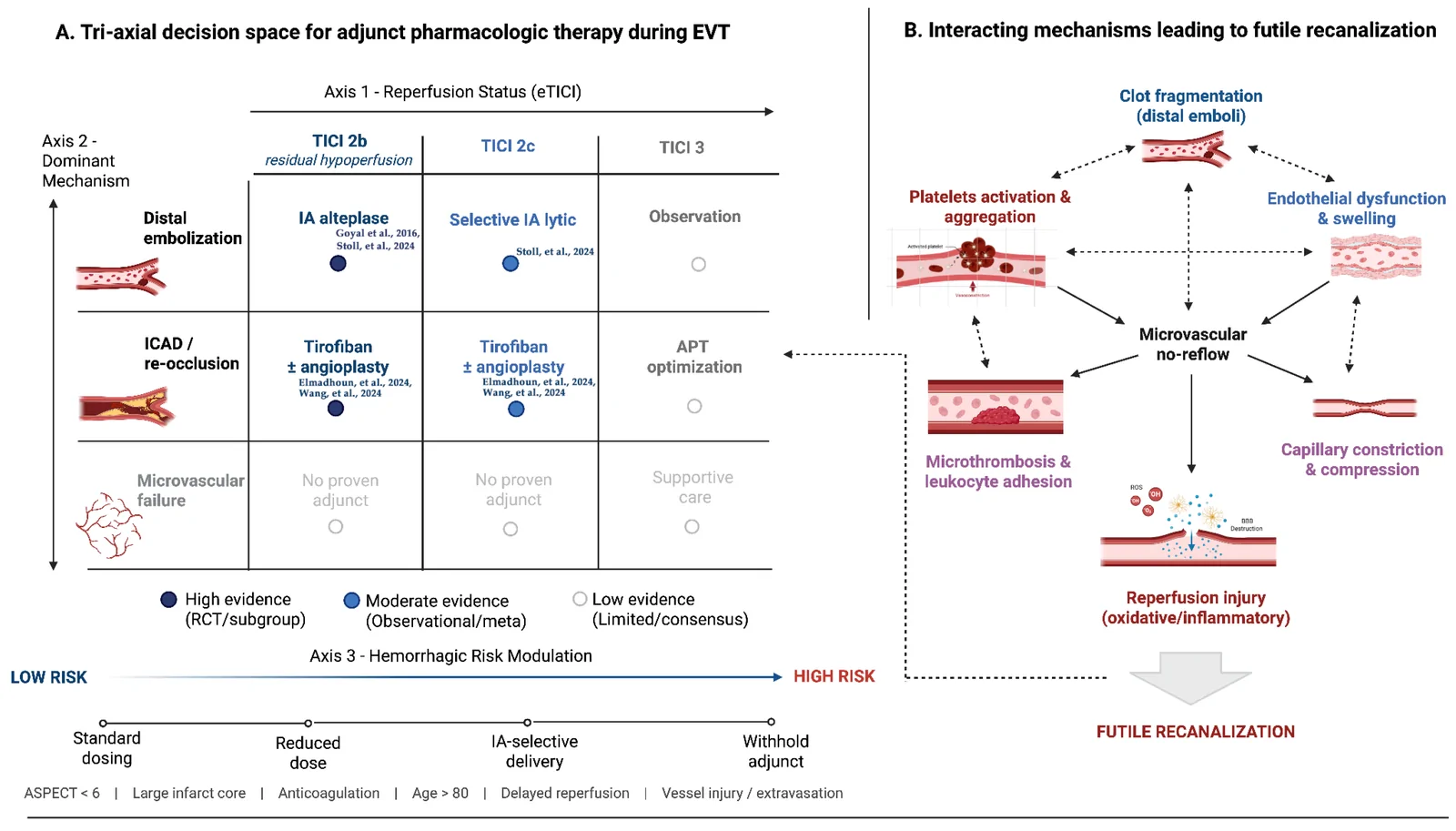
Decision algorithm for adjunct pharmacologic therapy during EVT. Proposed tri-axial framework integrating angiographic reperfusion status [eTICI grade], stroke mechanism [cardioembolic, intracranial atherosclerotic disease, or distal embolic/microvascular dysfunction], and hemorrhagic risk profile to guide selection of adjunct intra-arterial pharmacologic therapy after endovascular thrombectomy [1,2,4,5].

A schematic representation of the proposed mechanistic-procedural decision framework is provided in Figure 2. This framework is intended as a hypothesis-generating clinical model rather than a validated treatment algorithm. Its ability to improve patient selection, treatment efficacy, or clinical outcomes compared with current practice remains to be established prospectively.

### 5.2. Clinical Scenarios

Scenario A: Incomplete Reperfusion e[TICI 2b]—Intra-Arterial Thrombolytics. In patients with eTICI 2b50 reperfusion and acceptable bleeding risk, intra-arterial alteplase represents a potential adjunctive strategy supported by the CHOICE trial, although prospective confirmation of optimal patient selection and dosing remains necessary [15]. A meta-analysis showed better functional outcomes [OR 1.37; 95% CI 1.01–1.86] at no extra mortality costs [50].

Scenario B: Distal Embolization—Microcatheter-Directed Thrombolytics. A new branch occlusion during thrombectomy gives the impression of clot fragmentation plus peripheral migration. However, microcatheter-directed thrombolytics provide anatomical targeting that guide-catheter delivery cannot replicate. Available evidence remains predominantly observational; therefore, microcatheter-directed thrombolysis should be regarded as an investigational rescue strategy, with dosing individualized according to angiographic findings and bleeding risk [50,53].

Scenario C: ICAD or Re-occlusion—Antiplatelet Strategy. The residual fixed stenosis, in-stent re-occlusion, or eccentric plaque all provide evidence of ICAD. Intravenous tirofiban [10 μg/kg bolus + 0.1 μg/kg/min × 24 h] represents a mechanistically plausible adjunctive strategy in selected patients with ICAD-related re-occlusion or platelet-rich thrombus. The RESCUE BT subgroup analysis showed that patients treated secondarily with tirofiban had considerably better outcomes [aOR 1.68; 95% CI 1.11–2.56; *p* = 0.02] with no increase in symptomatic hemorrhage [7.1% for both groups] [52]. Mediation analysis showed that reduced thrombectomy passes helped to achieve 20% of the benefit. A meta-analysis reported similar findings: rescue therapy in ICAD-LVO resulted in a higher proportion of good outcomes [OR 3.19; 95% CI 1.91–5.32] and lower mortality rates [OR 0.35; 95% CI 0.16–0.76] [54].

Scenario D: High Hemorrhagic Risk—Conservative Approach. ASPECTS < 6, a prior large infarct, anticoagulation, or blood vessel perforation: these features substantially increase hemorrhagic risk and generally favor avoidance of pharmacologic thrombolysis [51,52]. The conservative management model of blood pressure control supplemented with diligent monitoring is a suitably safe approach. When antiplatelet therapy remains necessary, lower doses or intra-arterial delivery may reduce systemic exposure [54]. Notably, patients with ICAD and stenosis ≥ 70% carry a one-year recurrent stroke risk exceeding 20%; in this subgroup, antiplatelet intensification may be justified even at borderline hemorrhagic risk, provided delivery is intra-arterial and dosing is appropriately reduced [51]. Across all four scenarios, therapy selection must be contextualized within the broader evidence landscape and individualized to the patient’s procedural and clinical profile—a principle that underpins the framework’s integration with existing guidelines.

### 5.3. Integration with Existing Evidence

Current AHA/ASA guidelines provide no specific recommendations for adjunct intra-arterial pharmacotherapy during thrombectomy, reflecting the limited and heterogeneous evidence base. The proposed framework does not supersede clinical judgment but rather operationalizes existing mechanistic knowledge into a testable decision algorithm. However, the framework remains conceptual and has not been prospectively validated as a patient-selection strategy. In particular, no completed trial has established that imaging-guided selection based on residual hypoperfusion or no-reflow improves outcomes compared with current angiography-guided practice. Importantly, the pathophysiology of ICAD provides strong rationale for antiplatelet strategies: plaque rupture with in situ thrombosis, artery-to-artery embolization, hemodynamic injury, and branch occlusive disease are the primary mechanisms by which ICAD causes stroke. High-resolution vessel wall MRI may help distinguish ICAD from other stenosing arteriopathies and identify high-risk plaque features such as enhancement and intraplaque hemorrhage, potentially informing pre-procedural planning [51]. Until prospective validation is completed, application of adjunct pharmacotherapy should be restricted to experienced centers with rigorous hemorrhage monitoring. The growing body of evidence from randomized trials [CHOICE, RESCUE BT], post hoc analyses, and meta-analyses supports the potential efficacy of selected mechanism-directed adjunct strategies; however, the strength of evidence differs substantially between interventions, with intra-arterial thrombolysis supported by randomized trial data and antiplatelet rescue strategies in ICAD remaining more dependent on subgroup, observational, and post hoc evidence [50,52,54]. The proposed mechanistic–procedural framework and corresponding adjunct pharmacologic strategies are summarized in Table 1.

## 6. Practical Procedural Considerations

The procedural integration of adjunct pharmacologic therapy during mechanical thrombectomy [MT] remains highly individualized and is typically guided by angiographic findings, clot behavior, reperfusion quality, and perceived hemorrhagic risk rather than rigid treatment algorithms. Across contemporary practice, adjunctive agents are most commonly considered in the setting of incomplete reperfusion, distal embolization, persistent thrombus despite multiple passes, or suspected underlying intracranial atherosclerotic disease with a tendency toward rapid re-occlusion [56,57,58].

Dosing strategies vary substantially between institutions and published series, reflecting the absence of standardized protocols and the heterogeneity of clinical scenarios. For intra-arterial fibrinolytics, low-dose alteplase administration is most frequently described and is often delivered in incremental aliquots totaling approximately 12–20 mg depending on angiographic response and bleeding risk [15,59]. Similar variability has been reported with urokinase-based approaches [60]. Glycoprotein IIb/IIIa inhibitors, particularly tirofiban, are generally administered as low-dose intra-arterial boluses followed in selected cases by short intravenous infusions to maintain vessel patency in the setting of residual stenosis or platelet-rich thrombus [25]. Importantly, most reports emphasize titration according to procedural response rather than predetermined targets. Accordingly, these dosing approaches should not be interpreted as standardized evidence-based regimens, but rather as ranges reported in clinical studies and practice.

The timing of adjunct pharmacologic administration is equally complex. Intra-procedural delivery is typically employed as a rescue strategy when mechanical thrombectomy alone yields suboptimal reperfusion, particularly after repeated passes or in the presence of distal thromboembolic fragments inaccessible to device retrieval [61,62]. In such circumstances, local pharmacologic therapy may facilitate microvascular reperfusion while minimizing additional endothelial trauma from repeated instrumentation. Conversely, post-recanalization administration is generally intended to stabilize reperfused territories at risk of re-occlusion or to improve distal perfusion despite apparent macrovascular reopening. The CHOICE trial and subsequent observational analyses have renewed interest in post-reperfusion intra-arterial thrombolytic administration, particularly following near-complete or complete reperfusion [15].

Delivery technique represents another important procedural variable. Administration through the guide catheter allows rapid infusion with minimal interruption to workflow and may be sufficient when diffuse distal embolic burden is suspected [63]. However, selective delivery through a microcatheter positioned closer to the residual thrombus or distal vascular territory offers greater local drug concentration and potentially reduced systemic exposure [64]. Microcatheter-based administration is particularly attractive for focal distal occlusions, branch emboli, or persistent slow-flow phenomena beyond the reach of thrombectomy devices. Nonetheless, excessive distal catheter manipulation in fragile or ischemic vessels may itself contribute to endothelial injury or perforator compromise, necessitating careful procedural judgment [65,66].

Safety considerations remain central to patient selection and procedural decision-making. Hemorrhagic transformation risk is influenced by infarct core size, blood–brain barrier disruption, number of thrombectomy passes, concomitant intravenous thrombolysis, and procedural duration [67,68]. Consequently, many operators perform immediate intraprocedural imaging such as flat-panel CT or, when available, post-procedural dual-energy CT, to rule out significant contrast staining, evolving parenchymal hematoma, or extensive infarction before proceeding with adjunctive pharmacologic escalation [69].

Risk mitigation strategies generally favor conservative dosing, gradual infusion, and avoidance of aggressive pharmacologic rescue in patients with extensive infarct burden or clear hemorrhagic susceptibility. Particular caution is warranted after multiple device passes, vessel perforation, severe hypertension, or when substantial blood–brain barrier injury is suspected. Current evidence from observational studies and meta-analyses suggests that carefully selected adjunctive intra-arterial therapy does not appear to substantially increase symptomatic intracranial hemorrhage rates; however, the quality of evidence remains limited and significant uncertainty persists regarding optimal patient selection and treatment thresholds.

## 7. Future Research Agenda

The biological rationale for adjunct pharmacotherapy during EVT is increasingly supported, yet clinical confirmation has remained inconsistent. Heterogeneous populations, mechanism–treatment mismatch, timing, and endpoint limitations may contribute to neutral findings, although limited intrinsic efficacy of individual agents cannot be excluded. Four priorities define the research agenda required to resolve these uncertainties. These mechanisms should not be regarded as a fixed biological sequence or hierarchy; their relative contribution is likely to vary according to stroke etiology, ischemic duration, collateral status, reperfusion timing, and the extent and quality of tissue reperfusion.

### 7.1. Precision Trial Design: Enriching by Mechanism and Reperfusion Status

The most consequential reform available to the field is restriction of enrollment to mechanism-specific, reperfusion-stratified populations. Despite achieving angiographic success in more than 80% of cases, EVT fails to produce functional independence in more than half of patients, a gap driven not by proximal occlusion status but by residual thrombus burden and microvascular compromise [1]. A meta-analysis of RCTs confirmed that intra-arterial thrombolytics confer the greatest mRS 0–1 benefit in eTICI 2b50–2c rather than TICI 3 populations [57], and the STAIR XIII Consortium emphasized mechanism-enriched enrollment as an important strategy for generating more informative and actionable evidence [30]. Parallel enrichment is needed for etiology-defined cohorts: ICAD-LVO carries re-occlusion rates approaching 50% after mechanical retrieval [70] and the RESCUE BT secondary analysis suggests potential tirofiban benefit in selected patients with ICAD [52], while the OPTIMISTIC trial demonstrated that pre-procedural tirofiban improved first-pass recanalization in a broader LVO population excluding atrial fibrillation [71]—hypothesis-generating signals that warrant confirmatory trials using prospectively defined and validated criteria for ICAD phenotyping, potentially incorporating vessel-wall MRI.

### 7.2. Imaging-Guided Pharmacotherapy: Defining and Targeting No-Reflow

The no-reflow phenomenon—persistent microvascular hypoperfusion despite macrovascular recanalization—represents perhaps the most clinically urgent target for imaging-guided intervention. A pivotal 2025 pooled analysis of the EXTEND-IA trials showed that tissue-level no-reflow despite eTICI 2c–3 recanalization produced outcomes equivalent to failed thrombectomy, fundamentally reframing the reperfusion–outcome gap [11]. Post-thrombectomy perfusion imaging reliably identifies these areas of persistent hypoperfusion and correlates independently with clinical outcome [72], and Prospective studies have also demonstrated no-reflow among patients achieving angiographic eTICI 2b–3 reperfusion, consistent with the definition-dependent range described in Section 3.2 [9]. Incorporating CT perfusion-defined persistent hypoperfusion at 24 h as a co-primary endpoint alongside 90-day mRS [30] could provide a biologically sensitive outcome measure and may improve the efficiency of future trials. Early-phase studies should first establish the feasibility, safety, imaging thresholds, and biological response of such phenotype-selected interventions before progression to adequately powered trials using validated clinical outcomes such as 90-day functional status.

### 7.3. Drug–Device Synergy: Redefining the Combined Endpoint

Current adjunct trials treat the device and drug as sequential rather than synergistic interventions, an assumption the evidence no longer supports. BRIDGE-TNK [NEJM 2025] demonstrated that IV tenecteplase before thrombectomy improved 90-day functional independence [52.9% vs. 44.1%, *p* = 0.04], building on EXTEND-IA TNK’s demonstration that pre-procedural tenecteplase reduces thrombus burden at the time of device passage [73,74]. A target trial emulation further confirmed the functional advantage of bridging tenecteplase independent of reperfusion grade achieved [75]. Future trials should prospectively define combined endpoints capturing both eTICI grade and perfusion-defined tissue reperfusion at 24 h, and evaluate microcatheter-directed intra-arterial delivery as a pharmacokinetically precise adjunct strategy [76].

### 7.4. Personalized EVT: Integrating Clot Composition and Stroke Etiology

Thrombus biology varies systematically across stroke etiologies in ways that directly determine pharmacologic susceptibility. STRIP registry data demonstrate that cardioembolic and cryptogenic thrombi are fibrin-dominant, while ICAD thrombi are platelet-rich [77]—implying that fibrinolytic augmentation is biologically appropriate for the former and antiplatelet adjuncts for the latter. CT-based radiomics can now discriminate fibrin-dominant from RBC-dominant thrombi on non-contrast admission imaging with histopathologically validated accuracy [78], and dual-energy CT adds a further layer of spectral characterization [79]. Machine learning integration of these biomarkers could potentially enable prospective, etiology-stratified pharmacologic assignment at the point of care, although this approach remains investigational and has not yet been prospectively tested in completed clinical trials [77,80]. Accordingly, the current decision framework emphasizes established imaging and procedural parameters, while biomarker integration should be considered a future refinement once candidate biomarkers are prospectively validated for mechanistic phenotyping and treatment selection [24,30,31]. Circulating biomarkers, including D-dimer and markers of inflammation, endothelial dysfunction, and platelet activation, may provide complementary biological information and could eventually support phenotypic classification alongside imaging; however, their ability to identify treatment-responsive mechanisms or guide adjunctive therapy remains unvalidated.

### 7.5. Synthesis: Toward a Pharmacologically Augmented EVT Paradigm

These four priorities converge on a single imperative: moving from population-level to patient-level trial design, from angiographic to biological endpoints, and from empirical to mechanism-matched pharmacologic assignment, a direction formally endorsed by the STAIR XIII Consortium in 2026 [30,31]. The mixed results of the thrombolysis trial landscape positive signals from BRIDGE-TNK and EXTEND-IA TNK against null results from other studies are not a verdict against adjunct pharmacology. They may, in part, reflect methodological limitations of trials that enrolled heterogeneous populations and relied primarily on global functional outcomes to detect biology-level effects. Addressing these methodological limitations through mechanism-enriched enrollment, imaging-defined endpoints, pre-procedural clot characterization, and optimized drug–device timing may provide a more informative approach to evaluating adjunct pharmacology. Pharmacologically augmented reperfusion guided by mechanism-specific procedural phenotypes represents a promising and testable direction for future endovascular stroke research. The principal completed clinical trials evaluating pharmacologic augmentation of EVT are summarized in Table 2.

## 8. Conclusions

The convergence of mechanistic, procedural, and clinical evidence indicates that EVT alone cannot fully address the complex biological constraints that determine functional recovery after ischemic stroke. The current paradigm—prioritizing macrovascular recanalization while treating adjunctive pharmacotherapy as an afterthought—has produced a plateau in outcomes despite technical advances in thrombectomy devices and techniques.

The tri-axial decision framework proposed here offers a path forward by operationalizing the precision principle: matching pharmacologic strategy to the specific procedural phenotype defined by reperfusion status, stroke etiology, and hemorrhagic risk. Rather than asking whether adjunct therapy “works” as a universal adjunct, clinicians and trialists should ask which therapy, for which patient, at which time point, and under which reperfusion conditions. This reframing suggests that neutral meta-analyses may reflect treatment heterogeneity rather than uniform futility, supporting stratification rather than abandonment of adjunctive approaches.

Several practical implications emerge. First, routine collection of stroke etiology, eTICI grade, and hemorrhagic risk stratification should become standard in both clinical practice and future trial design, enabling real-time application of the framework. Second, the differential outcomes of CHOICE [positive in eTICI ≥2b50] versus POST-TNK/POST-UK [neutral in eTICI 2c–3] demonstrate that reperfusion grade is not merely a prognostic marker but a treatment-effect modifier—a distinction that should inform both bedside decisions and future trial designs. Third, the framework highlights evidence gaps that should prioritize future research: the optimal IA thrombolytic dose and agent in incomplete reperfusion, the role of glycoprotein IIb/IIIa inhibitors in ICAD-related re-occlusion, and the integration of perfusion imaging to identify no-reflow in real time. Intra-arterial thrombolysis after EVT has randomized evidence suggesting potential benefit in selected patients, particularly those with incomplete reperfusion, whereas glycoprotein IIb/IIIa inhibition for ICAD-related re-occlusion and microcatheter-directed thrombolysis for distal embolization remain supported predominantly by subgroup, observational, or mechanistic evidence.

The proposed framework remains hypothesis-generating and requires prospective validation before it can inform clinical treatment algorithms. Future studies should evaluate mechanism-specific pharmacologic strategies using imaging-defined tissue reperfusion and clinically meaningful outcomes. Although this review focuses on adjunct pharmacotherapy during EVT, the underlying concept of tissue-level reperfusion may ultimately extend beyond patients eligible for thrombectomy or intravenous thrombolysis, including selected patients with medium- or distal-vessel occlusions, particularly in light of recent evidence suggesting no improvement in functional recovery with mechanical thrombectomy over medical therapy alone in this population [81]; such applications remain outside the present scope and require independent evaluation.

## Figures and Tables

**Table 1:** Mechanism-guided framework for adjunct pharmacologic therapy during endovascular thrombectomy.

Scenario	Mechanism	Angiographic Signature	Adjunct Therapy	Evidence Level	Clinical Applicability	Research Priority	Hemorrhagic Risk	Reference
A: Incomplete Reperfusion	Residual distal thrombus/microvascular obstruction	eTICI 2b50–2b67, sluggish distal flow fields	IA alteplase [0.225 mg/kg] over 15–30 min	Moderate [CHOICE RCT; meta-analyses]	Potentially applicable in selected patients; supported by randomized evidence	Prospective validation of eTICI-based selection and optimal IA thrombolytic dose/agent	Low-to-Moderate	[15,50]
B: Distal Embolization	Thrombus fragmentation during mechanical retrieval	New filling defect in distal branch not present on baseline angiogram	Microcatheter-Directed IA alteplase [0.1–0.2 mg/kg]	Low [Case series; retrospective registries]	Investigational; not established for routine use	Prospective randomized evaluation of microcatheter-directed thrombolysis and optimal dosing	Low-to-Moderate	[50,53]
C: ICAD with Re-occlusion	Platelet-mediated rethrombosis at the stenotic site	Focal residual stenosis ≥ 70%, recurrent re-occlusion after passes	IV Tirofiban [10 µg/kg bolus + 0.1 µg/kg/min × 24 h] +/− angioplasty/stenting	Low–Moderate [post hoc/subgroup and observational evidence]	Promising but investigational; selective use based on procedural context	Prospective ICAD-specific trials to establish efficacy, optimal agent/dose, and patient selection	Moderate	[52,54]
D: High Hemorrhagic Risk	Established parenchymal injury or bleeding predisposition	ASPECTS < 6, parenchymal hematoma on pre-MT imaging, contrast extravasation	Withhold all adjunct pharmacotherapy	Guideline/consensus principles; limited direct evidence	Avoid adjunct pharmacotherapy; conservative management favored	Validation of hemorrhagic-risk thresholds and safe criteria for withholding or modifying adjunct therapy	High	[52,55]

Abbreviations: eTICI = expanded Thrombolysis in Cerebral Infarction scale; ICAD = intracranial atherosclerotic disease; IA = intra-arterial; IV = intravenous; MT = mechanical thrombectomy; ASPECTS = Alberta Stroke Program Early CT Score; DWI = diffusion-weighted imaging; CT-P = CT perfusion; SBP = systolic blood pressure; APT = antiplatelet therapy; RCT = randomized controlled trial; GP = glycoprotein; IVT = intravenous thrombolysis; aOR = adjusted odds ratio; OR = odds ratio.

**Table 2:** Key clinical studies informing adjunct pharmacologic strategies during EVT.

Trial/Study Name	Agent/Intervention	Route	Target Population	Principle Outcome/Endpoint	Phase/Design	Key Hypothesis/Mechanism	Reference
EXTEND-IA pooled analysis [EXTEND-IA trials pooled cohort]	Observational [no agent]	—	Patients with eTICI 2c-3 recanalization post-EVT	90-day functional outcome by tissue-level perfusion status	Pooled analysis	Tissue-level no-reflow negates clinical benefit of successful macrovascular recanalization	[11]
RESCUE BT [secondary analysis]	Tirofiban [IV bolus + infusion]	IV	ICAD-LVO with successful reperfusion [eTICI 2b50-3]	90-day functional independence [mRS 0–1]	Phase 3 RCT	Antiplatelet adjunct reduces re-occlusion in platelet-rich ICAD thrombi	[52]
OPTIMISTIC	Tirofiban [IV]	IV	LVO [excluding atrial fibrillation] before EVT	First-pass recanalization; mRS 0–2 at 90 days	Phase 3 RCT	Pre-procedural antiplatelet priming increases rapid recanalization in ICAD	[71]
BRIDGE-TNK	Tenecteplase 0.25 mg/kg	IV [pre-EVT]	LVO within 4.5 h of onset	mRS 0–2 at 90 days	Phase 3 RCT	Pre-thrombectomy thrombolysis reduces thrombus burden and improves functional outcome	[73]
EXTEND-IA TNK	Tenecteplase 0.25 mg/kg vs. alteplase	IV [pre-EVT]	LVO eligible for EVT within 4.5 h	Pre-EVT recanalization rate; mRS 0–1 at 90 days	Phase 3 RCT	Tenecteplase achieves higher pre-catheter reperfusion than alteplase	[74]

## Data Availability

No new data were created or analyzed in this study. Data sharing is not applicable to this article.

## Abbreviations

The following abbreviations are used in this manuscript:

AIS	Acute Ischemic Stroke
AHA/ASA	American Heart Association/American Stroke Association
aOR	Adjusted Odds Ratio
APT	Antiplatelet Therapy
ASPECTS	Alberta Stroke Program Early CT Score
BBB	Blood–Brain Barrier
CT	Computed Tomography
CT-P	Computed Tomography Perfusion
DWI	Diffusion-Weighted Imaging
eTICI	Expanded Thrombolysis in Cerebral Infarction
EVT	Endovascular Thrombectomy
GP	Glycoprotein
GPVI	Glycoprotein VI
HIR	Hypoperfusion Intensity Ratio
IA	Intra-Arterial
ICAD	Intracranial Atherosclerotic Disease
ICH	Intracranial Hemorrhage
IL-6	Interleukin-6
IV	Intravenous
IVT	Intravenous Thrombolysis
LVO	Large Vessel Occlusion
mRS	Modified Rankin Scale
MRI	Magnetic Resonance Imaging
mTICI	Modified Thrombolysis in Cerebral Infarction
MT	Mechanical Thrombectomy
OR	Odds Ratio
RCT	Randomized Controlled Trial
RCTs	Randomized Controlled Trials
ROS	Reactive Oxygen Species
SBP	Systolic Blood Pressure
sICH	Symptomatic Intracranial Hemorrhage
STAIR	Stroke Therapy Academic Industry Roundtable
SUR1	Sulfonylurea Receptor 1
TICI	Thrombolysis in Cerebral Infarction
TLR4	Toll-Like Receptor 4
Tmax	Time-to-Maximum of the Residue Function
TNK	Tenecteplase
